# Reversal of tenofovir induced nephrotoxicity: case reports of two patients

**DOI:** 10.11604/pamj.2017.27.126.10033

**Published:** 2017-06-16

**Authors:** Fatuma Some, Mathew Koech, Emily Chesire, Gabriel Kigen

**Affiliations:** 1 Department of Medicine, Moi University School of Medicine, P.O. Box 4606, Eldoret, Kenya; 2 Moi Teaching and Referral Hospital, P.O. Box 3, Eldoret, Kenya; 3 Department of Child Health and Pediatrics, Moi University School of Medicine, P.O. Box 4606, Eldoret, Kenya; 4 Department of Pharmacology and Toxicology, Moi University School of Medicine, P.O. Box 4606, Eldoret, Kenya

**Keywords:** Case report, HIV/AIDs, tenofovir, nephrotoxicity, reversal

## Abstract

The use of the antiretroviral drug tenofovir has been associated with nephrotoxicity. However, the overall impact of this adverse effect has not comprehensively evaluated. Some researchers have reported that it is quite severe to warrant monitoring for renal toxicity, while others have concluded that the magnitude may not be that significant. We report two clinical cases seen in our renal clinic with high creatinine levels suggestive of nephrotoxicity who reverted back to normality upon withdrawal of tenofovir.

## Introduction

Tenofovir disoproxil fumarate (TDF), the first nucleotidic inhibitor of HIV reverse transcription, is now one of the most widely used antiretroviral drug. This is largely due to its high antiretroviral activity, relatively good metabolic profile and more importantly good compliance as it has a once-daily dosing. It has been co-formulated with emtricitabine (FTC) and efavirenz (EFV) to form a single fixed-dose combination (ATRIPLA^®^), which has greatly improved patient adherence [1], as it has a once-daily dosing. Another fixed dose combination of TDF, lamivudine (3TC) and EFV is commonly used in resource-limited settings. In fact, the World Health Organization (WHO) has recommended that TDF be included in the first line management of HIV/AIDS in both adults and adolescents, as well as children [2]. However, there are several published reports relating to TDF-induced renal toxicity [3, 4]. This has been attributed to the accumulation of TDF in the proximal renal tubular cells, leading to mitochondrial toxicity, and subsequent renal tubular acidosis leading to acute kidney injury [5, 6]. There have also been reports of patient recovery upon discontinuation of TDF [5]. We report two clinical cases seen in our renal clinic with high creatinine levels suggestive of nephrotoxicity who reverted back to normality upon withdrawal of TDF.

## Patient and observation

### Case 1

A 54-year-old patient with known HIV infection and on combined antiretroviral treatment (cART) consisting of TDF, 3TC and EFV was referred to hospital with a 4-day history of abdominal pains, decreased appetite and abdominal bloating. Two weeks earlier he had been vomiting, and had taken herbal medicines without improvement. The abdominal pains were associated with passage of loose stool and fever. At the referring hospital, his blood pressure (BP) was 121/60 mmHg and he had a fever at 37.8°C. He was oliguric with urine output of 55ml over 24 hours, with no evidence of haematuria and body swelling. He reported no previous illnesses apart from HIV infection diagnosed 3 months earlier. At the referral center, his BP was 70/40 mmHg; pulse rate 116 beats per minute and temperature 37.4°C. He had mild pallor and minimal edema. Systemic examination was unremarkable. On admission, his serum creatinine was 1361μmol/l, urea concentration 30mmol/l and the electrolyte concentrations were: sodium 126mmol/l, potassium 5.8mmol/l, chloride 94mmol/l, and calcium 1.91mmol/l. He also had mild anaemia with a hemoglobin concentration of 9.7g/dL, a white cell count of 20,000/μl, and platelets 329,000/μl. Ultrasound showed no urinary obstruction or calculi, the kidneys were echogenic with loss of corticomedullary differentiation; the right kidney measured 11.1 by 5.3cm, the left 12.2 by 5.25cm. The bladder was normal and prostate mildly enlarged. The rest of the abdomen was normal. He was started on haemodialysis and the cART regimen changed to ZDV, renal-dosed 3TC and EFV and discharged after 3 sessions. He was then followed up as an outpatient. After three weeks, he was readmitted with symptomatic anaemia, transfused with one unit of blood, dialysed and discharged after a brief stay. Five weeks after the first discharge (two weeks after the second) he was seen at the dialysis unit with a creatinine level of 388μmol/l and dialysis was withheld. At the next visit in two weeks, his creatinine levels had dropped to 113μmol/l, and dialysis was discontinued.

### Case 2

A 53-year-old male known with HIV infection since 2010 was admitted to hospital in April 2014 with symptoms of vomiting and abdominal pains for three days prior. It was associated with nausea and the vomitus contained food, was non-blood stained and occurred 3-4 times a day. He also complained of dizziness, some cough and had recently noted that his urine output had reduced without change in colour. He however had no history of diarrhoea or fever. He had been started on first line drugs; zidovudine (AZT), 3TC and EFV in 2010 and also put on cotrimoxazole prophylaxis of 960mg daily. He had a history of tuberculosis treatment the same year, for which he completed medication. In 2012, he was switched to second line treatment of ritonavir-boosted lopinavir (LPV/r), 3TC and TDF due to drug failure. His most recent viral load in 2013 was undetectable. On admission, he was very pale with oral thrush, was severely wasted and volume depleted; and had no oedema. His blood pressure was 100/60 and temperature 36.1 degrees. His systemic exam was otherwise unremarkable. Laboratory tests revealed the following: white cell count of 2,350/μl, hemoglobin of 3.8g/dL and a platelet count of 353,000/μl. Creatinine was 559μmol/l, urea 55mmol/l, potassium 4.5mmol/l and sodium of 114mmol/l. He had hepatitis B surface antigen positivity. He was put on antibiotics, fluconazole, rehydrated and transfused. TDF was discontinued and replaced by ABC, 3TC was renal dosed, LPV/r continued and cotrimoxazole dose halved. On the third day, the creatinine level was 541μmol/l, urea 35mmol/l, potassium 2.5mmol/l, chloride 86mmol/l and sodium122mmol/l. He was managed for hypokalemia. Over the next few days the creatinine levels reduced progressively and at discharge the creatinine level was down to 216μmol/l and urea to 33mmol/l. He was followed up and a month later, his renal function had normalized.

## Discussion

We report two cases of patients with renal toxicity that was likely associated with tenofovir. Both patients presented with acute kidney injury (AKI) and upon withdrawal of TDF and subsequent follow up the kidney function normalized. The first case describes a patient who had just been diagnosed with HIV infection and developed AKI that necessitated haemodialysis as part management, three months after initiation of cART which included TDF. The second developed injury after two years of treatment. There are many reported studies associating TDF with renal toxicity, including in children [3, 4, 7]. Some of the predisposing factors that have been identified include: dose and duration of treatment, low CD4 count, advanced age and low body weight, co-prescription of didanosine or boosted protease inhibitor, pre-existing chronic kidney disease (CKD) and associated diabetes mellitus [8–10]. The adverse effect has also been reported to be more prevalent in patients from low income communities [11]. The onset for the development of renal toxicity has been reported to be between five months [12] and ten years [13]. Pretreatment with melatonin and use of antioxidants with mitochondria-targeted properties has been postulated to prevent proximal tubular mitochondrial against TDF damage [10]. Our two cases developed AKI from a combination of factors, which included TDF. Case 1 had a history of precedent vomiting, use of herbal medicines and closer to admission, a history of loose stools and fever and a documented episode of hypotension. He could have had a pre-renal renal failure, acute interstitial nephritis or acute tubular necrosis, diagnoses which were not conclusively made. Case 2 had a history of vomiting, was fluid depleted and hypotensive. The high ratio of urea to creatinine is typical of a pre-renal failure but an element of acute tubular necrosis seemed to have played a role as the recovery was more protracted. It is our opinion that the presence of TDF might have “primed” the kidney for injury in these 2 cases. The TDF had made the kidney vulnerable to insult and made it more likely for the patient to develop AKI. The overall impact of the TDF-induced renal toxicity has not been conclusively evaluated. Whereas some researchers have suggested that it is quite severe to warrant monitoring for renal toxicity [14], others have concluded that the magnitude may not be that significant [15, 16]. In addition, there have also been reports of reversal of renal toxicity upon TDF withdrawal [17, 18]. Some drugs, including rosiglitazone have even been reported to reverse the nephrotoxicity [19], although other researchers have reported that the reversal is not complete in infected men [20]. This has generated debate on the severity of the adverse effect and the general safety of the long-term use of TDF [9].

## Conclusion

We report two cases of patients with high creatinine levels suggestive of nephrotoxicity whose kidney function normalized upon withdrawal of TDF and subsequent follow up. Further research should be conducted, perhaps including pharmacogenomic studies from different sites in order to evaluate the magnitude of this adverse effect that has been associated with this important drug.

## Competing interests

The authors declare no competing interest.
